# The cervicovaginal microbiome as a candidate correlate and potential predictor of HPV clearance and treatment response after HSIL therapy: a scoping review

**DOI:** 10.3389/fonc.2026.1972845

**Published:** 2026-10-22

**Authors:** Benjamin Mwesige, Bridget Angucia, Langanani Mbodi, Absalom Mwazha, Nixon Niyonzima, Judith Mwansa-Kambafwile, Robert F. Breiman, Henry Zakumumpa, Admire Chikandiwa

**Affiliations:** 1 Uganda Cancer Institute, Kampala, Uganda; 2 Department of Obsetrics and Gynecology Oncology Unit, University of Witwatersrand, Johannesburg, South Africa; 3 Discipline of Anatomical Pathology, School of Medicine, College of Health Sciences, Universitry of KwaZulu-Natal, Durban, South Africa; 4 National Health Laboratory Services, Johannesburg, South Africa; 5 Department of Global Health, Rollins School of Public Health, Emory University, Atlanta, GA, United States; 6 School of Public Health, Makerere University, Kampala, Uganda; 7 Wits Planetary Health Research, Faculty of Health Sciences and affiliate of IDORI, University of Witwatersrand, Johannesburg, South Africa

**Keywords:** cervicovaginal microbiome, high-grade squamous intraepithelial lesion, human papillomavirus clearance, *Lactobacillus*, loop electrosurgical excision procedure

## Abstract

**Background:**

Persistent high-risk human papillomavirus (hrHPV) infection causes virtually all high-grade squamous intraepithelial lesions (HSIL) and cervical cancer, but only a minority progress, implicating host and microenvironmental cofactors. The cervicovaginal microbiome (CVM), particularly Lactobacillus-dominated communities, may influence hrHPV persistence, clearance, and response to HSIL treatment.

**Objective:**

To map evidence linking the CVM with hrHPV clearance and outcomes after HSIL treatment, including loop electrosurgical excision procedure (LEEP) and conization, using PRISMA-ScR and Arksey and O’Malley frameworks.

**Methods:**

Literature on cervicovaginal microbiota, community state types (CSTs), HPV natural history, HSIL/CIN treatment, probiotics, microbiome-directed interventions, and predictive biomarkers was systematically searched and narratively synthesized using a Population-Concept-Context framework.

**Results:**

Sixty-six sources were charted. *Lactobacillus crispatus*-dominated communities (CST-I) were associated with hrHPV clearance and favorable post-treatment outcomes, whereas CST-IV, bacterial vaginosis, and increased alpha-diversity were associated with hrHPV persistence, greater HSIL severity, and treatment failure. Proposed mechanisms include lactic acid-mediated pH regulation, D-lactic acid signaling, and modulation of viral oncoproteins and immune responses. Excision altered the CVM, with restoration of Lactobacillus dominance accompanying viral clearance. Microbial-signature machine-learning models showed promising but preliminary predictive performance. Oral and intravaginal probiotics showed inconsistent but generally favorable trends, with strongest evidence for intravaginal *L. crispatus* transplantation. Gut microbiome diversity may also influence chemoradiotherapy outcomes in invasive cervical cancer.

**Conclusion:**

The CVM is a promising predictor of hrHPV clearance and HSIL treatment response. However, methodological heterogeneity, small cohorts, and limited prospective evidence restrict clinical translation. Large prospective studies and rigorously designed trials are needed to validate predictive signatures and determine whether microbiome profiling or targeted modulation can improve HSIL management and outcomes.

## 1 Introduction

Cervical cancer remains a major global health burden. According to GLOBOCAN 2022 estimates, cervical cancer accounted for approximately 662, 301 new cases and 348, 874 deaths worldwide, ranking eighth in incidence and ninth in mortality among all cancers in women (1–3). Marked regional disparities persist, with the highest age-standardized incidence and mortality concentrated in sub-Saharan Africa, and projections suggesting that many low- and middle-Human-Development-Index countries will not meet the World Health Organization’s 2030 elimination targets (90% HPV vaccination coverage, 70% screening coverage, 90% treatment of precancer and cancer) without accelerated intervention (4). Persistent infection with high-risk human papillomavirus (hrHPV), most commonly genotypes 16 and 18, is the necessary etiological driver of high-grade squamous intraepithelial lesion (HSIL, encompassing cervical intraepithelial neoplasia grade 2 and 3 [CIN2/3]) and invasive cervical cancer (5–7).

Despite the near-universal detection of hrHPV DNA in HSIL and cancer, the great majority of incident hrHPV infections are transient: 70-90% clear spontaneously within one to two years, and only a small fraction of persistent infections progress through HSIL to invasive disease (8, 9). This discordance between viral exposure and disease outcome has focused attention on host and local microenvironmental cofactors that modify the natural history of hrHPV infection. Among these, the cervicovaginal microbiome (CVM) — the community of bacteria, and to a lesser extent fungi and viruses, colonizing the vaginal and ectocervical mucosa — has emerged over the past 15 years as a biologically plausible and increasingly well-characterized modulator of hrHPV acquisition, persistence, clearance, and lesion progression (10–12).

The foundational work of Ravel and colleagues established that the vaginal microbiota of reproductive-age women clusters into a limited number of community state types (CSTs): four are typically dominated by a single Lactobacillus species (CST-I, Lactobacillus crispatus, hereafter abbreviated L. crispatus; CST-II, L. gasseri; CST-III, L. iners; CST-V, L. jensenii), while CST-IV comprises a polymicrobial, largely anaerobic community with depleted Lactobacillus abundance and is further subdivided into CST-IVA and CST-IVB (13, 14). Subsequent studies refined this taxonomy across ethnically diverse populations and identified additional variants, including Bifidobacterium- and Gardnerella-subgroup-dominated communities (15, 16). Lactobacillus-dominated, and particularly L. crispatus-dominated, communities maintain a low vaginal pH through lactic acid production, support mucosal barrier integrity, and are associated with reduced susceptibility to sexually transmitted pathogens including HPV, whereas CST-IV and bacterial vaginosis (BV) states are characterized by chronic low-grade inflammation and epithelial barrier disruption (17–21).

Excisional and ablative treatment of HSIL — most commonly the loop electrosurgical excision procedure (LEEP), but also cold-knife conization and laser procedures — is highly effective, achieving hrHPV clearance and histological cure in the large majority of women. However, 5-20% of treated women experience persistent or recurrent hrHPV infection and disease, and identifying which women are at risk before or shortly after treatment remains an unmet clinical need (22–24). Because excisional treatment itself physically and physiologically remodels the cervicovaginal niche, and because pretreatment microbiota composition has been linked to post-treatment outcome, the CVM represents a candidate biomarker — and potentially a modifiable target — for predicting and improving treatment response (22, 25, 26).

The evidence linking the cervicovaginal microbiome to hrHPV natural history and HSIL treatment outcomes is rapidly expanding but remains highly heterogeneous in study design, methodology, and clinical focus. The literature encompasses cross-sectional association studies, longitudinal cohorts, mechanistic *in vitro* investigations, probiotic intervention trials, and emerging machine-learning biomarker models. Consequently, a scoping review represents the most appropriate synthesis framework, enabling systematic mapping of the breadth, nature, and methodological diversity of available evidence while identifying critical knowledge gaps and emerging research priorities beyond the scope of a narrowly defined intervention-focused systematic review.

This review aims to (1) map the extent and nature of evidence linking the CVM to hrHPV clearance across the natural history of infection (2); characterize how the CVM changes with, and predicts response to, HSIL-directed excisional therapy (3); summarize proposed mechanisms linking microbiota composition to HPV oncogenesis and viral clearance (4); catalogue microbiome-targeted and probiotic interventions trialed to date; and (5) identify methodological gaps to inform future research and eventual clinical translation.

## 2 Materials and methods

This scoping review was conducted in accordance with the methodological framework originally proposed by Arksey and O’Malley (27) and subsequently refined by Levac, Colquhoun, and colleagues (28, 29), and is reported following the Preferred Reporting Items for Systematic Reviews and Meta-Analyses extension for Scoping Reviews (PRISMA-ScR) 20-item checklist (30). Consistent with Joanna Briggs Institute guidance for scoping reviews (31), no formal risk-of-bias-weighted synthesis or pooled effect estimate was undertaken; instead, evidence was systematically charted and mapped to characterize the breadth and distribution of the field. No prior protocol registration was required for this narrative synthesis, and the review question was intentionally framed broadly to capture the full scope of relevant evidence. It should be emphasized that, while this review adheres to the PRISMA-ScR reporting checklist and follows a structured, reproducible search strategy, it did not employ independent dual-reviewer screening, *a priori* protocol registration, or formal inter-rater agreement assessment; the process is therefore best characterized as a structured, PRISMA-ScR-concordant evidence-mapping exercise rather than a fully systematic scoping review in the strictest methodological sense, and conclusions should be interpreted accordingly.

### 2.1 Research question and PCC framework

The review question was framed using the Population-Concept-Context (PCC) structure recommended for scoping reviews (30, 31) Population: Women of reproductive and peri-/post-menopausal age with hrHPV infection, cervical intraepithelial neoplasia, HSIL, or cervical cancer, including cohorts undergoing excisional or ablative HSIL treatment.

• Concept: Composition, diversity, and dynamics of the cervicovaginal (vaginal and cervical) microbiome, including community state types, Lactobacillus dominance, bacterial vaginosis, and microbial metabolites, in relation to hrHPV acquisition, persistence, clearance, HSIL/CIN severity, and post-treatment outcome.
• Context: Observational (cross-sectional, cohort, case-control), interventional (probiotic and microbiome-modulating trials), and mechanistic (*in vitro*, immunological, metabolomic) studies conducted in clinical and translational research settings globally, without restriction on country income level.

### 2.2 Search strategy and sources

A structured, iterative search was conducted across biomedical literature databases and indexing platforms (including PubMed/MEDLINE-indexed content, Scopus- and Embase-indexed journals, and publisher platforms such as Frontiers, MDPI, Nature Portfolio, BMC/Springer Nature, PLOS, and Wiley) using combinations of the following key terms and their variants: “cervicovaginal microbiome”, “vaginal microbiota”, “community state type”, “Lactobacillus crispatus”, “bacterial vaginosis”, “human papillomavirus”, “HPV clearance”, “HPV persistence”, “cervical intraepithelial neoplasia”, “high-grade squamous intraepithelial lesion”, “loop electrosurgical excision procedure”, “conization”, “probiotic”, and “cervical cancer treatment response”. The complete PubMed/MEDLINE search string, together with its adapted forms for Scopus, Embase, and the publisher-specific search interfaces listed above, to permit independent replication of the search; in brief, the core PubMed strategy combined MeSH terms and free-text variants for the exposure (e.g., (“vaginal microbiome”[tiab] OR “cervicovaginal microbiome”[tiab] OR “vaginal microbiota”[tiab] OR “Lactobacillus”[tiab] OR “community state type”[tiab] OR “bacterial vaginosis”[tiab]) AND (“papillomavirus infections”[MeSH] OR “HPV”[tiab] OR “human papillomavirus”[tiab] OR “cervical intraepithelial neoplasia”[tiab] OR “CIN”[tiab] OR “high-grade squamous intraepithelial lesion”[tiab] OR “HSIL”[tiab] OR “LEEP”[tiab] OR “conization”[tiab] OR “cervical cancer”[tiab])), with results limited to human studies and further screened by title/abstract and full text against the eligibility criteria below. Reference lists of key reviews and systematic reviews were hand-searched for additional eligible sources (10, 32–35). Searches were not restricted by publication date but prioritized literature from 2011 (coinciding with the foundational CST taxonomy) onward (13), supplemented by seminal earlier mechanistic and epidemiological work where relevant.

### 2.3 Eligibility criteria

Sources were included if they reported original data or synthesized evidence on cervicovaginal or vaginal microbiota composition in relation to HPV natural history, cervical dysplasia, or HSIL/CIN treatment outcomes, or if they described relevant methodological, epidemiological, or mechanistic context necessary to interpret this evidence (e.g., scoping/systematic review methodology, global cervical cancer epidemiology, CST taxonomy, immunological mechanisms of HPV oncogenesis). Studies were excluded if they addressed the vaginal microbiome exclusively in contexts unrelated to HPV or cervical neoplasia (e.g., isolated preterm birth or infertility outcomes) unless cited for methodological or physiological background. Conference abstracts without extractable data, non-peer-reviewed opinion pieces, and studies unavailable in English were excluded where identified. The English-language restriction was adopted primarily for pragmatic and resource reasons: the review team lacked professional translation capacity for full-text screening and data extraction in other languages, and reliable machine translation of specialized microbiological and histopathological terminology (e.g., CST nomenclature, Nugent scoring, histological grading) was judged too error-prone to support accurate charting. This restriction is nonetheless a substantive limitation rather than a purely procedural one, because a considerable proportion of the primary LEEP/conization-microbiome literature originates from Chinese-language sources; its implications for the completeness and generalizability of this synthesis are discussed further under Limitations.

### 2.4 Data charting and synthesis

For each included source, data were charted on: study design, population and sample size, microbial profiling method (e.g., 16S rRNA gene sequencing, qPCR microarray, Nugent scoring, metagenomics), CST or dysbiosis classification used, HPV/HSIL outcome measured, and principal findings. Charted data were grouped into thematic domains reflecting the review’s objectives: (i) CVM physiology and taxonomy; (ii) CVM and hrHPV acquisition/clearance; (iii) mechanistic pathways linking microbiota to HPV oncogenesis and immune clearance; (iv) CVM dynamics and predictive value around HSIL excisional/ablative treatment; (v) predictive biomarker and machine-learning models; (vi) probiotic and microbiome-directed interventions; and (vii) gut microbiome and treatment response in invasive disease. Findings were synthesized narratively with a summary evidence table, consistent with the descriptive, non-pooled analytic approach appropriate to scoping reviews (27, 28, 30).

## 3 Results

Sixty-six unique sources met the eligibility criteria and were charted; **Table 1** provides the characteristics (study design, population/focus, and thematic category) for every included source. Because most sources address more than one thematic domain — for example, a mechanistic *in vitro* study may also report cross-sectional clearance data, and a taxonomy paper may also serve as methodological background — the per-category counts below are not mutually exclusive and their sum exceeds the total number of included sources: systematic and narrative reviews (n≈12), cross-sectional and case-control microbiome studies (n≈22), prospective cohort studies around HSIL treatment (n≈8), randomized or controlled probiotic intervention trials (n≈6), mechanistic/immunological and *in vitro* studies (n≈8), predictive/machine-learning modeling studies (n≈4), gut microbiome-chemoradiotherapy studies (n≈4), epidemiological/burden-of-disease reports (n≈6), and methodological/menopause-physiology sources (n≈10). Of these 66 sources, only a small subset directly evaluated hrHPV clearance or lesion outcome within the peri-treatment window around LEEP, conization, or ablative therapy (five sources: refs. 22-26, detailed in Section 3.4 and **Table 2**); the remaining sources characterize the broader biology, taxonomy, immunology, or non-HSIL-specific treatment context of the CVM-hrHPV relationship and are included as necessary interpretive background rather than as direct evidence on post-treatment outcomes. This distinction between direct treatment-outcome evidence and broader contextual evidence is maintained throughout the synthesis below and is flagged explicitly for each source in **Table 1**. The evidence is synthesized below by thematic domain.

**Table 1:** Characteristics of all included sources (n=66).

Ref	Study	Design/N	Thematic category	Direct HSIL post-treatment evidence?
(1)	Hakim et al., 2025	Narrative review	Epidemiology/burden	Background
(2)	Son et al., 2026	Epidemiological analysis (GLOBOCAN)	Epidemiology/burden	Background
(3)	Wu J et al., 2025	Epidemiological analysis (GLOBOCAN)	Epidemiology/burden	Background
(4)	Zhu et al., 2024	Epidemiological analysis	Epidemiology/burden	Background
(5)	Li Y et al., 2025	Bioinformatic/mechanistic analysis	Mechanistic (oncogenesis)	Background
(6)	Zhang J et al., 2025	Epidemiological analysis	Epidemiology/burden	Background
(7)	Jouya et al., 2026	Narrative review	Epidemiology/burden	Background
(8)	Chao et al., 2021	Cross-sectional, n=272	Predictive biomarker (lesion severity)	Background
(9)	Linz et al., 2026	Longitudinal cohort	CVM and hrHPV persistence	Background
(10)	Dai W et al., 2021	Narrative review	Review (CVM and HPV clearance)	Background
(11)	Leon-Gomez & Romero, 2025	Narrative review	Review (CVM/metagenomics)	Background
(12)	Chen J et al., 2025	Narrative review	Review/mechanistic (immune modulation)	Background
(13)	Ravel et al., 2011	Cross-sectional, n=396	Taxonomy (CST foundational)	Background
(14)	Gajer et al., 2012	Longitudinal cohort	Taxonomy (temporal dynamics)	Background
(15)	Doyle et al., 2018	Cross-sectional	Taxonomy (population-specific)	Background
(16)	Albert et al., 2015	Cross-sectional	Taxonomy (population-specific)	Background
(17)	Shen et al., 2022	Narrative review	Taxonomy/physiology	Background
(18)	Liu P et al., 2023	Narrative review	Probiotic/mechanistic	Background
(19)	France et al., 2022	Narrative review	Taxonomy	Background
(20)	Smith & Ravel, 2017	Narrative review	Physiology	Background
(21)	De Seta et al., 2022	Cross-sectional/mechanistic	Immune mechanism (CST-immune network)	Background
(22)	Caselli et al., 2020	Prospective, n=41 (LEEP)	CVM and HSIL treatment outcome	Direct
(23)	Liang et al., 2021	Case-control, n=108 (conization)	CVM and HSIL treatment outcome	Direct
(24)	Wiik et al., 2019	Prospective cohort, n=89 vs 100 (LEEP)	CVM and HSIL treatment outcome	Direct
(25)	Janicka-Kośnik et al., 2021	Registered systematic review protocol (PROSPERO)	CVM and HSIL treatment outcome (protocol)	Direct
(26)	Zhang H et al., 2018	Prospective, n=26 (LEEP)	CVM and HSIL treatment outcome	Direct
(27)	Arksey & O’Malley, 2005	Methodological framework paper	Methodology	Background
(28)	Colquhoun et al., 2014	Methodological framework paper	Methodology	Background
(29)	Daudt et al., 2013	Methodological framework paper	Methodology	Background
(30)	Tricco et al., 2018	Reporting guideline (PRISMA-ScR)	Methodology	Background
(31)	Peters et al., 2020	Methodological guidance (JBI)	Methodology	Background
(32)	Mancilla et al., 2024	Systematic review	Review (Latina population)	Background
(33)	Pai et al., 2025	Systematic review	Review (vaginal/gut microbiota)	Background
(34)	Alizhan et al., 2025	Narrative review	Review/mechanistic (physiology)	Background
(35)	Incognito et al., 2025	Systematic review	Review (diversity and cancer)	Background
(36)	Dai W et al., 2026	Cross-sectional + functional validation	Mechanistic (D-lactic acid)	Background
(37)	Lee YH et al., 2020	Cross-sectional, n=66 (random-forest model)	Predictive/machine-learning	Background
(38)	Lin F et al., 2025	Narrative review	Physiology (menopause)	Background
(39)	Lin W et al., 2025	Cross-sectional	Physiology (menopause)	Background
(40)	Shardell et al., 2021	Cross-sectional	Physiology (menopause)	Background
(41)	Nieto et al., 2025	Narrative review	Physiology (menopause)	Background
(42)	Ovalle, 2025	Narrative review	Physiology (menopause)	Background
(43)	Di Paola et al., 2017	Cohort	CVM and hrHPV persistence	Background
(44)	Guo et al., 2012	Cohort	CVM and hrHPV persistence	Background
(45)	Avsaroglu et al., 2023	Cohort	CVM and hrHPV persistence	Background
(46)	Kongrtay et al., 2025	Prospective cohort	CVM and hrHPV acquisition	Background
(47)	Nicolò et al., 2023	*In vitro*	Mechanistic (oncoprotein modulation)	Background
(48)	Liu H et al., 2022	Metagenomic cross-sectional	Mechanistic (oncogene expression)	Background
(49)	Delgado-Diaz et al., 2020	*In vitro*	Mechanistic (immune)	Background
(50)	Wang W & Yu 2026	Narrative review	Mechanistic (metabolites)	Background
(51)	Dai W et al., 2022	Cross-sectional/mechanistic	Mechanistic (immune, Langerhans cells)	Background
(52)	Rahma et al., 2014	Clinical trial (immunotherapy)	Mechanistic (immune)	Background
(53)	Liu Y et al., 2024	RCT pilot, n=100 (L. crispatus chen-01)	Probiotic/microbiome-directed intervention	Background
(54)	Ou et al., 2019	RCT, n=121 (oral probiotic)	Probiotic/microbiome-directed intervention	Background
(55)	Verhoeven et al., 2013	Controlled pilot (oral probiotic)	Probiotic/microbiome-directed intervention	Background
(56)	Sims et al., 2021	Prospective, n=51 (chemoradiation)	Gut microbiome and treatment response	Background
(57)	Rosas Cancio-Suárez et al., 2025	Cross-sectional	CVM and HSIL development	Background
(58)	Bokulich et al., 2022	Multi-omics modeling	Predictive/machine-learning	Background
(59)	Gomes et al., 2026	Perspective review	Predictive/machine-learning	Background
(60)	Wang Z et al., 2025	Mendelian randomization analysis	Mechanistic (genetic/immune)	Background
(61)	Şahin & Ceylan, 2025	Retrospective cohort (oral probiotic)	Probiotic/microbiome-directed intervention	Background
(62)	Susetiati et al., 2025	Systematic review/meta-analysis	Probiotic/microbiome-directed intervention (review)	Background
(63)	Kęczkowska et al., 2025	Narrative review	Review (Lactobacillus and clearance)	Background
(64)	El Alam et al., 2021	Prospective (chemoradiotherapy)	Gut microbiome and treatment response	Background
(65)	Wen et al., 2025	Systematic review/meta-analysis	Review (gut/cervical/vaginal microbiota)	Background
(66)	Wu X et al., 2026	Narrative review	Review/mechanistic (CRT resistance)	Background

Full characteristics of every source included in this scoping review, addressing the request for a comprehensive rather than selective evidence table. Thematic categories are non-mutually-exclusive (see Results); the final column flags the small subset of sources providing direct evidence on hrHPV clearance or lesion outcome specifically within the peri-treatment window around LEEP, conization, or ablative therapy for HSIL, as distinct from sources addressing the broader biology of the cervicovaginal microbiome and HPV natural history.

**Table 2:** Selected studies linking the cervicovaginal microbiome to HSIL treatment response and HPV clearance.

Study focus	Design/n	Key CVM finding	Outcome measure	Ref
LEEP + cytokine profiling	Prospective, n=41 (Ferrara, Italy)	CST-IV/non-L. crispatus at baseline predicted persistence & recurrence	hrHPV status, CIN recurrence at 6 mo	(22)
LEEP, 16S rRNA	Prospective, n=26 (China)	Diversity fell after LEEP; pre-LEEP Lactobacillus depletion linked to persistence	hrHPV clearance at 3 mo	(26)
Cervical conization	Case-control, n=108 (Xinjiang, China)	Persistent HPV linked to BV- and AV-associated anaerobes, biofilm phenotype	hrHPV persistence post-conization	(23)
LEEP, cone depth	Prospective cohort, n=89 vs 100 controls (Norway)	Microbiota shifted post-LEEP; associations with cone depth explored	HPV persistence, cytokine/microbiota change	(24)
Conization, CST changes	Prospective, n=85 HSIL (Caselli et al. design)	CST-IV decreased significantly in hrHPV-cleared but not persistent group	CST distribution pre/post-surgery	(23, 25)
Baseline CST and persistence	Cohort, 55 HPV+/17 HPV- (Italy)	CST-IV present in 43% of persistence group vs 7.4% of clearance group	1-year HPV persistence vs clearance	(43)
Vaginal probiotic transplantation	RCT pilot, n=100	Intravaginal L. crispatus chen-01 reduced viral load, increased clearance, restructured VMB	hrHPV clearance, viral load, cytology	(53)
Oral probiotic (GR-1/RC-14)	RCT, n=121	No significant difference in HPV clearance; improved smear quality	hrHPV clearance, smear adequacy	(54)
Oral probiotic drink pilot	Controlled pilot, n=54	Trend toward higher clearance of cytological abnormalities with probiotic	Cytology and HPV clearance at 6 mo	(55)
Vaginal microbiome ML biomarkers	Cross-sectional, n=272	Stenotrophomonas, Streptococcus, Pseudomonas up; Dialister, Bifidobacterium down in HSIL	HSIL prediction thresholds	(8)
Random-forest CIN severity model	Cross-sectional, n=66	33-species panel, AUC 0.952 for CIN2+ vs CIN1-; L. iners top predictor	Diagnostic accuracy for CIN severity	(37)
Gut microbiome & chemoradiation	Prospective, n=51 (cervical cancer)	Higher gut diversity independently associated with survival and CD4+ T-cell infiltration	Overall survival, immune infiltration	(56)

CST, community state type; VMB, vaginal microbiota; LEEP, loop electrosurgical excision procedure; AV, aerobic vaginitis; ML, machine learning; RCT, randomized controlled trial.

### 3.1 Physiology and taxonomy of the cervicovaginal microbiome

The healthy reproductive-age CVM is characterized by relative simplicity and Lactobacillus dominance, in contrast to the high diversity typical of other mucosal niches such as the gut (17, 20). Ravel et al.’s landmark 16S rRNA sequencing study of 396 asymptomatic North American women established five principal community state types, four Lactobacillus-dominated (CST-I/L. crispatus, CST-II/L. gasseri, CST-III/L. iners, CST-V/L. jensenii) and one polymicrobial, anaerobe-rich type (CST-IV) associated with bacterial vaginosis and adverse reproductive outcomes (13). Longitudinal sampling subsequently demonstrated that CSTs are dynamic, with transitions occurring over days to weeks in relation to menses, sexual activity, and hygiene practices, and that CST-IV itself comprises at least two subtypes (IV-A, moderate anaerobe burden with residual L. iners; IV-B, high bacterial-vaginosis-associated bacteria [BVAB] burden) (14, 18). Ethnically diverse cohorts, including Canadian and sub-Saharan African populations, have confirmed the broad applicability of this taxonomy while highlighting population-specific variation in CST prevalence and additional Gardnerella-subgroup or Bifidobacterium-dominated profiles (15, 16, 21).

Mechanistically, L. crispatus dominance is distinguished by high production of D (–)-lactic acid (relative to L-lactic acid produced preferentially by host cells and other Lactobacillus species), yielding a lower, more protective vaginal pH and greater mucosal barrier integrity than communities dominated by L. iners or by CST-IV anaerobes (34, 36). L. iners, although a Lactobacillus species, occupies an intermediate ecological position: it is more commonly found in transitional or dysbiotic states, produces less protective lactic acid, and has been inconsistently associated with hrHPV clearance across studies (32, 37).

The CVM is not static across the reproductive lifespan. Estrogen-dependent glycogen deposition in the vaginal epithelium supports Lactobacillus proliferation during the reproductive years; declining estrogen at menopause reduces glycogen availability, promotes Lactobacillus depletion, and increases the prevalence of CST-IV-like, higher-diversity communities and clinical bacterial vaginosis — a phenomenon sometimes termed the “menopause paradox” of declining dominance alongside increasing richness (38–42). These age- and hormone-related shifts are clinically relevant because HSIL treatment and HPV natural history span pre-, peri-, and post-menopausal populations, and because vaginal estrogen or hormone therapy can partially restore Lactobacillus dominance (40, 41).

### 3.2 The cervicovaginal microbiome and hrHPV acquisition, persistence, and clearance

A consistent theme across cross-sectional and longitudinal studies is that hrHPV-positive women, and particularly those with CIN or cervical cancer, exhibit higher CVM alpha-diversity and lower relative Lactobacillus abundance than hrHPV-negative controls (1, 10, 11). A systematic review focused on Latina populations identified 42 unique cervicovaginal taxa associated with HPV infection, dysplasia, or cancer across 25 included studies, and confirmed that L. crispatus was consistently associated with HPV resistance and clearance, while L. iners was not and was more frequently detected alongside bacterial vaginosis (32). A broader systematic review of CVM diversity and Lactobacillus profiles in invasive cervical cancer similarly found that non-Lactobacillus-dominant and high-diversity profiles were overrepresented in cancer cases relative to controls (35), and a 2025 systematic review of vaginal and gut microbiota across the HPV-CIN-cancer continuum reinforced that altered microbiota, rather than HPV infection alone, tracks with disease progression and clearance (33).

Prospective evidence directly addressing viral clearance versus persistence, rather than cross-sectional case-control comparisons, is more limited but concordant. In an Italian cohort of hrHPV-positive women followed for one year, CST-IV was present at baseline in 43% of women whose infection persisted versus only 7.4% of women who cleared the virus, with Atopobium spp. significantly enriched in the persistence group (43). A large longitudinal case-control analysis of BV status assessed on Pap smear microscopy similarly found higher hrHPV persistence and cytological progression rates among BV-positive women, with Lactobacillus-dominant flora consistently associated with favorable clearance outcomes across two independent cohorts (9), corroborating earlier work by Guo and colleagues showing higher BV prevalence in a persistent-HPV cohort (11.2%) than in a clearing cohort (5.0%) (44). A separate cohort study found that a decrease in cervicovaginal lactobacilli was independently associated with HPV persistence after adjustment for age and menopausal status (odds ratio 2.67) (45), and a prospective cohort from Kazakhstan linked disrupted vaginal microbiota — including Chlamydia trachomatis co-infection and Mycoplasma/Ureaplasma colonization — to increased HPV acquisition risk among non-vaccinated women (46).

Mechanistic work at the bacterium-oncogene interface has begun to explain these associations. Co-culture experiments using HPV16-transformed SiHa cells demonstrated that L. crispatus and L. gasseri modulate basal E6/E7 oncogene expression and downstream p53/pRb oncosuppressor production, whereas dysbiosis-associated species (including Gardnerella and Megasphaera) had contrasting, sometimes oncogene-promoting, effects, and L. iners and L. jensenii produced mild induction of E7 expression relative to the null effect of L. crispatus (34, 47). A bacterial cocktail of L. crispatus, L. gasseri, and L. jensenii reduced expression of E6, E7, cyclin A, and CDK2 *in vitro*, suggesting a mechanistic basis for reduced malignant transformation risk under Lactobacillus dominance (34). Metagenomic analysis of cervical swabs across the HPV-CIN-cancer spectrum found progressively increasing microbial diversity and decreasing Lactobacillus abundance paralleling rising E6/E7 oncogene expression, with genera such as Sneathia, Leptotrichia, and Pseudomonas positively correlated with oncogene expression (48).

### 3.3 Proposed immunological and metabolic mechanisms linking the CVM to HPV clearance

It is important to distinguish, at the outset of this subsection, between what is comparatively well established and what remains proposed or hypothesis-generating. The epidemiological association between Lactobacillus-dominated, particularly L. crispatus-dominated, communities and favorable hrHPV outcomes is reasonably well established across multiple independent observational cohorts. In contrast, the specific molecular and immunological pathways summarized below — miRNA induction, Langerhans cell modulation, and cytokine/chemokine signaling cascades linking microbial metabolites to HPV oncogene suppression — derive predominantly from single *in vitro* or small cross-sectional studies and should be regarded as proposed, mechanistically plausible pathways rather than confirmed causal mechanisms *in vivo*. Beyond direct microbe-oncogene interactions, the CVM shapes local innate and adaptive immunity in ways plausibly relevant to hrHPV clearance. Lactic acid, particularly the D-isomer preferentially produced by L. crispatus, elicits anti-inflammatory responses from cervicovaginal epithelial cells and suppresses pro-inflammatory mediators associated with mucosal susceptibility to viral acquisition, whereas short-chain fatty acids and metabolite profiles characteristic of BV-like, non-optimal microbiota dysregulate epithelial immune responses, increasing TNF-alpha production while suppressing protective chemokines such as RANTES and IP-10 (49, 50). *In vitro* work has further shown that lactic acid can induce miR-744 expression, which in turn suppresses HPV E6/E7 oncogene expression both directly and via ARHGAP5-mediated pathways, offering a molecular explanation for epidemiological associations between Lactobacillus dominance and lesion regression (50). A cross-sectional multi-omics study integrating 16S rRNA sequencing, metabolomics, and cervical transcriptomics found that L. crispatus-derived D-lactic acid correlated with distinct cervical gene expression signatures and with reduced likelihood of high-grade lesions among HPV-positive women, reinforcing a mechanistic chain from microbial metabolite to host transcriptional response to lesion severity (36).

Antigen-presenting Langerhans cells (LCs) of the cervical epithelium represent a further mechanistic node: HPV oncoproteins are known to suppress LC antigen uptake and presentation, and cervicovaginal microbiota composition has been proposed to modulate LC activation state and HPV-specific cellular immunity, offering a route by which dysbiosis could impair viral clearance independent of direct oncogene modulation (51). Complementary evidence from dendritic-cell-based immunotherapy studies in advanced cervical cancer, in which HPV16 E6/E7-peptide-pulsed dendritic cells elicited measurable antigen-specific immune responses in the majority of evaluable patients, underscores the biological plausibility that an immunologically permissive local microenvironment — of which the microbiome is one determinant — is central to effective anti-HPV immunity (52). At the community level, the “Vaginal CST Microbiome-Immune Network” concept proposes that each CST is associated with a distinct local immune-protein signature, with L. crispatus-dominant communities linked to immune tolerance and non-Lactobacillus communities linked to pro-inflammatory activation, a framework directly applicable to understanding differential hrHPV outcomes across CSTs (21). Toll-like receptor signaling perturbation by HPV E6/E7 proteins, which reduces interferon-alpha secretion in response to pathogen-associated molecular patterns, may further compound the effects of a pro-inflammatory dysbiotic microenvironment on viral persistence (34).

### 3.4 The CVM as a predictor of and response marker for HSIL excisional and ablative treatment

The evidence most directly relevant to this review’s central question concerns whether pretreatment CVM composition predicts hrHPV clearance and lesion resolution after HSIL therapy, and how the CVM itself changes across the treatment episode. A prospective observational study of women undergoing LEEP for CIN2/3 in Italy found that four patients with persistent HPV infection and CIN2/3 recurrence at 6-month follow-up exhibited a CST-I (L. crispatus-dominant) profile at baseline but were characterized by unusually high loads of Gardnerella vaginalis, Ureaplasma parvum, and Candida albicans compared to the broader CST-I group, with minimal post-treatment microbiota shift, suggesting that co-colonization with opportunistic organisms — not CST label alone — may better flag treatment-failure risk within apparently favorable communities (22).

A Chinese prospective cohort using 16S rRNA sequencing before and three months after LEEP for CIN2/3 documented an overall decrease in cervical microbial diversity following treatment, consistent with mechanical debridement of the ectocervical dysbiotic niche during excision (26). A larger case-control study from Xinjiang, China, comparing HPV-positive women without conization, HPV-positive women after conization with persistent infection, and HPV-negative women after conization, found that postoperative persistent HPV infection was associated with both classic BV-associated anaerobes (Gardnerella, Atopobium) and aerobic-vaginitis-associated pathogens (Enterococcus and related genera), together with anaerobic and biofilm-forming bacterial phenotypes, implicating biofilm persistence as a possible mechanism of post-surgical viral non-clearance (23). A Norwegian cohort study of 89 women undergoing LEEP, compared with 100 cytologically normal references, examined the relationship between cervical microbiota, cone depth, and HPV persistence, adding data on how the physical extent of excision intersects with microbiota-mediated clearance (24).

A key mechanistic-clinical synthesis derives from a prospective comparison of the vaginal microbiota of 85 HSIL patients before and after conization, which found that surgical removal of the HPV-associated lesion was accompanied by a significant decrease in CST-IV prevalence specifically among women who subsequently cleared hrHPV, while CST-IV prevalence remained essentially unchanged in women whose infection persisted (23). This finding — that microbiome “reconstruction” toward Lactobacillus dominance parallels, and may be necessary for, durable viral clearance after excisional treatment — represents one of the most direct pieces of evidence supporting the CVM as both a predictor and a potential mediator of treatment response, rather than merely a bystander marker. A related analysis found that higher HPV16/18 viral load was more closely associated with CST-IV in women younger than 50 years, suggesting that age-stratified interpretation of microbiome-viral load relationships may be needed when using the CVM to risk-stratify HSIL patients around the time of treatment (23).

Together, this body of work — summarized in **Table 2** — supports three converging conclusions: (i) pretreatment CVM composition, and specifically CST-IV/dysbiotic status, is associated with a higher risk of post-excisional hrHPV persistence and lesion recurrence; (ii) excisional treatment itself measurably alters the CVM, generally reducing overall diversity; and (iii) the trajectory of post-treatment microbiome recovery toward Lactobacillus dominance, rather than a single time-point measurement, may carry the strongest predictive and mechanistic signal for durable clearance.

### 3.5 Predictive biomarkers and machine-learning models

A growing subset of the literature applies supervised machine-learning approaches to CVM sequencing data to predict lesion severity, and by extension, to explore feasibility for predicting treatment-relevant outcomes. A study of 272 women (83 HSIL, 86 HPV-positive without neoplasia, 103 HPV-negative controls) identified candidate 16S rDNA biomarkers — increased Stenotrophomonas, Streptococcus, and Pseudomonas, with decreased Dialister, Bifidobacterium, Faecalibacterium, and Bacteroides — that distinguished HSIL from lower-risk states, with taxon-specific abundance thresholds proposed as a potential adjunct to cervical cancer screening (8). A separate study built a random-forest classifier using a 33-species panel that distinguished CIN2+ from CIN1- with an area under the curve of 0.952, with L. iners identified as the single most impactful predictor — an intriguing finding given L. iners’ otherwise ambiguous status as an intermediate, only partially protective Lactobacillus species (37). More recent work applying differential abundance and functional pathway analyses (ANCOM-BC2, KEGG/EC enrichment) to cervical microbiota in the development of HSIL identified 37 KEGG orthologs and 17 enzyme-commission terms significantly associated with HSIL status, including pathways in glycine, serine, threonine, and sulfur metabolism, moving the field from purely compositional to functional biomarker discovery (57).

Multi-omics integration studies combining vaginal microbiome, pH, metabolome, and immunoproteome data using neural network and random-forest regression have identified the cervicovaginal metabolome as the strongest overall predictor of microenvironmental state relevant to neoplasia risk, ahead of microbiome composition alone, suggesting that future predictive models for treatment response may need to incorporate metabolomic and immunological layers rather than 16S data in isolation (58). A 2026 perspective review specifically proposes integrating vaginal microbiome signatures with HPV genotype, host DNA methylation markers, and clinical/behavioral factors into multi-modal artificial intelligence models for cervical cancer risk prediction, explicitly framing non-Lactobacillus-dominated and L. iners-rich communities as complementary biomarkers alongside conventional risk factors (59). A causal-inference approach using bidirectional Mendelian randomization has also begun to interrogate whether specific immune cell populations causally contribute to HSIL risk, complementing correlative microbiome-immune findings and pointing toward future integration of genetic, immunological, and microbial data streams (60). Notably, none of the machine-learning studies identified to date were trained or validated specifically on post-treatment (post-LEEP/conization) outcome data; existing models predict lesion severity or presence, not treatment response, representing a clear translational gap.

### 3.6 Probiotic and microbiome-directed interventions

If the CVM is causally, not merely correlatively, linked to hrHPV clearance, then microbiome-directed intervention should improve clearance rates — a hypothesis tested in a small but growing number of trials. An early controlled pilot study found that women with HPV-associated low-grade cytological abnormalities who consumed a daily oral probiotic drink had numerically, though not statistically significantly, higher rates of HPV clearance (29% vs 19%) and significantly higher clearance of cytological abnormalities (60% vs 31%, p=0.05) compared to untreated controls over six months (55). A subsequent randomized, double-blind, placebo-controlled trial of oral L. rhamnosus GR-1 and L. reuteri RC-14 in 121 women with genital hrHPV found no significant difference in hrHPV clearance rate between probiotic and placebo groups (58.1% vs 54.2%), although the probiotic group showed significantly reduced rates of mildly abnormal and unsatisfactory cervical smears at 6 months, and lower baseline viral load was the only independent predictor of clearance identified (54). A retrospective study of oral L. rhamnosus similarly explored its effect on hrHPV clearance and cervical smear outcomes, adding real-world observational data to the oral-probiotic evidence base (61).

In contrast, intravaginal probiotic transplantation — delivering live Lactobacillus directly to the site of infection rather than relying on oral administration and ascending colonization — has shown more consistent benefit. A prospective controlled pilot study of intravaginal L. crispatus chen-01 transplantation in 100 women with hrHPV infection found that the treatment group had significantly reduced HPV viral load, higher clearance rates, and improved vaginal inflammatory status without notable adverse effects, with 16S rRNA sequencing confirming that transplantation effectively reconstituted a Lactobacillus-dominant vaginal microbiota, providing a plausible causal mechanism for the clinical benefit observed (53). A systematic review and meta-analysis of Lactobacillus therapy (oral and vaginal, pooled) for cervical HPV infection found an overall trend toward benefit but noted substantial heterogeneity in strain, dose, delivery route, and outcome definition across included trials, precluding firm pooled conclusions and underscoring the need for standardized trial design (62). A complementary narrative review synthesizing molecular, *in vivo*, and clinical trial evidence on Lactobacillus-mediated HPV clearance similarly concluded that L. crispatus and L. gasseri exert protective effects through immune modulation, acidic pH maintenance, and competitive exclusion of pathogens, positioning selected-strain probiotics as a promising, though not yet validated, adjunct to conventional HSIL management (63).

No randomized trial identified in this review specifically tested probiotic or microbiome-modulating intervention as an adjunct to LEEP or conization to improve post-treatment hrHPV clearance or reduce recurrence — a second clear translational gap, distinct from, but related to, the absence of machine-learning models trained on post-treatment data noted above. A registered systematic review and meta-analysis protocol addressing CVM changes specifically after CIN treatment (PROSPERO CRD42021255805) was identified, indicating that the field recognizes this gap and that pooled quantitative synthesis of post-treatment microbiome data is anticipated as more primary studies accumulate (25).

### 3.7 Gut microbiome, chemoradiotherapy, and broader treatment response in cervical cancer

For women with invasive cervical cancer treated with chemoradiotherapy (CRT) rather than excisional therapy for HSIL, an analogous but mechanistically distinct literature has emerged linking gut, rather than purely cervicovaginal, microbiome diversity to treatment response and survival. In a prospective study of cervical cancer patients receiving definitive chemoradiation, higher gut microbiome diversity was independently associated with improved short- and long-term survival, and with increased tumor infiltration of CD4+ lymphocytes and activated (Ki67+, CD69+) T-cell subsets over the course of radiotherapy, suggesting that gut microbial diversity may potentiate radiation-induced antitumor immunity through mechanisms paralleling those described for immune checkpoint blockade in other cancers (56). A prospective study of the temporal dynamics of the gut microbiome during pelvic CRT for gynecologic cancers, including cervical cancer, found that gut diversity decreased during treatment and did not fully normalize by short-term follow-up, with antibiotic exposure as a significant confounder — highlighting the need for careful covariate adjustment in future microbiome-treatment-response studies (64). A systematic review and meta-analysis integrating gut, cervical, and vaginal microbiota data across cervical cancer studies confirmed that antineoplastic treatment (chemoradiotherapy, radical hysterectomy, and other modalities) measurably alters microbiota composition, and proposed an estrogen-mediated gut-vaginal axis, noting substantial taxonomic overlap between gut and vaginal communities, as a mechanistic bridge between these two anatomically distinct but physiologically linked microbial niches (65). Complementary work on microbiome-host interactions in locally advanced cervical cancer treated with CRT similarly concludes that dysbiosis is associated with reduced treatment efficacy and increased toxicity, while microbiome-targeted interventions — analogous to the probiotic strategies described above for HSIL — are proposed as a means of optimizing outcomes and mitigating treatment-related adverse effects, pending longitudinal studies integrating gut and vaginal microbiome data with immune infiltrates and patient-reported outcomes (66). A broader synthesis of the vaginal microbiome’s role in immune modulation in cervical cancer situates these treatment-response findings within the tumor microenvironment literature, emphasizing host-microbe interaction as a determinant of both carcinogenesis and therapeutic response across the disease spectrum from HSIL to invasive cancer (12).

Although this literature primarily concerns invasive cancer rather than HSIL per se, it is directly relevant to this review because it establishes proof-of-concept that microbiome diversity and composition — at both cervicovaginal and gut sites — causally or quasi-causally influence response to standard oncological therapy in the cervical cancer disease continuum, strengthening the biological plausibility that analogous relationships exist, and are clinically actionable, at the earlier HSIL/excisional-treatment stage that is the primary focus of this review.

## 4 Discussion

### 4.1 Synthesis of the evidence

This scoping review maps a rapidly maturing but still heterogeneous evidence base linking the cervicovaginal microbiome to hrHPV clearance and, more specifically, to response to HSIL-directed excisional and ablative treatment. Across observational, mechanistic, and interventional study designs, a consistent pattern emerges: Lactobacillus-dominated, and particularly L. crispatus-dominated (CST-I), communities are associated with more effective hrHPV clearance, lower HSIL severity, and better outcomes after LEEP or conization, while CST-IV, bacterial-vaginosis-associated, and high-diversity communities are associated with persistence, recurrence, and treatment failure (9, 22, 23, 32, 35, 43, 44). This pattern is biologically coherent with mechanistic data demonstrating that L. crispatus-derived D-lactic acid and low vaginal pH suppress HPV E6/E7 oncogene expression, promote anti-inflammatory epithelial responses, and support antigen-presenting cell function, whereas dysbiotic metabolite profiles and anaerobe overgrowth promote pro-inflammatory, oncogene-permissive conditions (21, 34, 36, 47, 49–51).

Extending this mechanistic reasoning specifically to the post-excisional setting, several complementary pathways plausibly explain why Lactobacillus dominance, and particularly L. crispatus dominance, is associated with more effective hrHPV clearance and fewer recurrences after LEEP or conization rather than only with clearance in the untreated natural history of infection. First, excisional treatment creates a transient cervical wound that must re-epithelialize, and the low-pH, D-lactic-acid-rich microenvironment sustained by L. crispatus limits colonization by anaerobic and biofilm-forming organisms (e.g., Gardnerella, Atopobium, Ureaplasma) that have been directly implicated in post-conization persistence; a Lactobacillus-dominant baseline or early post-treatment community may therefore reduce the biofilm-mediated reservoir effect proposed as a mechanism of post-surgical viral non-clearance. Second, the same anti-inflammatory, TNF-alpha-suppressing and RANTES/IP-10-preserving epithelial response elicited by D-lactic acid would be expected to support, rather than impair, the local immune surveillance required to clear residual HPV-infected keratinocytes at the wound margin during re-epithelialization, whereas the pro-inflammatory, barrier-disrupting profile of CST-IV communities could both delay healing and sustain a permissive niche for oncogene-expressing cells that escaped excision. Third, because miR-744 induction and ARHGAP5-mediated E6/E7 suppression have been linked *in vitro* to lactic acid exposure, restoration of Lactobacillus dominance in the weeks after treatment could actively suppress oncogene expression in any residual transformed cells, providing a molecular rationale for the empirical observation that CST-IV reduction specifically among women who cleared hrHPV, rather than pretreatment CST alone, tracked with durable clearance. Finally, Langerhans cell antigen-presentation capacity, which is impaired by HPV oncoproteins and modulated by local microbiota, may recover more effectively in a Lactobacillus-dominant post-treatment niche, supporting the cell-mediated immune response needed to eliminate any HPV-infected cells spared by excision. Taken together, these proposed mechanisms converge on a single, testable hypothesis — that the peri-treatment trajectory of microbiome recovery, not merely its pretreatment state, actively participates in determining treatment success — but it should be emphasized that this integrated mechanistic model has not yet been directly tested in a single study spanning microbiota, immune, and virological endpoints within the LEEP/conization window, and remains an important priority for future mechanistic-clinical research (see Future Research Priorities).

The mechanistic basis for this pattern is best understood as operating through several convergent pathways rather than a single mechanism, and each pathway has direct relevance to the peri-treatment window specifically, not only to hrHPV natural history in general. First, at the level of viral oncogene expression, L. crispatus-derived D (–)-lactic acid and the accompanying low vaginal pH suppress HPV E6/E7 oncoprotein expression and downstream p53/pRb turnover in co-culture systems, and a Lactobacillus cocktail (L. crispatus, L. gasseri, L. jensenii) further reduces cyclin A and CDK2 expression *in vitro* (34, 48); because E6/E7 suppression directly antagonizes the viral machinery that drives keratinocyte immortalization, sustained L. crispatus dominance after excision would be expected to lower the oncogenic pressure on any HPV-infected cells left behind at the margin. Second, at the level of local immunity, lactic acid signaling induces miR-744, which suppresses E6/E7 expression via ARHGAP5-mediated pathways, and elicits an anti-inflammatory, chemokine-preserving (RANTES/IP-10) epithelial response, in contrast to the TNF-alpha-driven, chemokine-suppressing profile associated with BV-like metabolites (50, 51); because durable hrHPV clearance ultimately depends on an effective local T-cell and antigen-presenting-cell response, and Langerhans cell antigen presentation is itself modulated by microbiota composition (21, 51), a favorable post-treatment immune milieu is plausibly necessary, not merely correlated, for clearing any residual infection after surgery. Third, and most specific to the treatment window, dysbiotic and biofilm-forming organisms (Gardnerella, Atopobium, Enterococcus, and related aerobic-vaginitis taxa) have been directly implicated in postoperative persistent hrHPV infection, with a biofilm phenotype proposed as a means by which non-Lactobacillus communities shield residual virus or infected keratinocytes from immune clearance and impede normal reepithelialization after excision (23); conversely, the observation that CST-IV prevalence fell significantly only among women who subsequently cleared hrHPV after conization, and remained essentially unchanged in women whose infection persisted, fits a model in which the excisional wound-healing process itself creates a window in which either Lactobacillus reconstitution (favoring clearance) or biofilm-associated dysbiosis (favoring persistence) becomes established, depending on the pre- and immediate post-treatment microbiota trajectory (23, 25). Taken together, these oncogene-suppressive, immunomodulatory, and anti-biofilm mechanisms provide a biologically coherent, multi-level explanation for why Lactobacillus dominance, and L. crispatus dominance in particular, tracks with more effective hrHPV clearance and better outcomes specifically after LEEP or conization, rather than reflecting a purely epiphenomenal association between microbiota and treatment response. It should be noted, as discussed further below, that much of this mechanistic evidence derives from *in vitro* or small cross-sectional studies rather than from mechanistic sampling performed within treatment cohorts themselves, so the chain from molecular mechanism to clinical treatment outcome remains inferential rather than directly demonstrated.

The most clinically important, and most directly relevant, finding for the treatment-response question posed by this review is that excisional treatment of HSIL is not microbiologically neutral: it measurably alters the cervicovaginal niche, generally reducing overall diversity, and the extent to which this remodeling favors Lactobacillus dominance — rather than simply the pretreatment CST label — appears to track with durable hrHPV clearance (23, 26). This suggests a dynamic, bidirectional relationship in which the microbiome both predicts and potentially participates in mediating treatment success, rather than functioning purely as a static pretreatment biomarker.

### 4.2 Clinical and translational implications

Three translational avenues emerge from this synthesis. First, pretreatment or early post-treatment CVM profiling — whether by targeted qPCR for CST classification, 16S rRNA sequencing, or emerging machine-learning-derived taxon panels;could in principle be incorporated into risk stratification for women undergoing LEEP or conization, flagging those with CST-IV or high-diversity profiles for closer post-treatment surveillance or adjunctive intervention (8, 37, 58). However, no validated, clinically deployable CVM-based risk score currently exists for this specific application, and the machine-learning models identified in this review were trained to predict lesion presence or severity rather than post-treatment clearance, limiting immediate clinical applicability.

Second, microbiome-directed intervention, particularly intravaginal Lactobacillus transplantation, which has shown more consistent benefit than oral probiotic administration; represents a plausible adjunctive strategy to accelerate hrHPV clearance either before or after excisional treatment (53, 62, 63). The absence of trials specifically testing probiotic adjuncts around the time of LEEP or conization represents the single clearest evidence gap identified in this review and should be prioritized in future randomized trial design, potentially using post-treatment hrHPV persistence at 6–12 months as the primary endpoint, informed by the existing PROSPERO-registered protocol for post-treatment CVM changes (25).

Third, the demonstrated relevance of gut microbiome diversity to chemoradiotherapy response in invasive cervical cancer raises the question that is not yet addressed in the HSIL-specific literature reviewed here; of whether gut microbiome status independently modulates outcomes after HSIL excisional treatment, given the estrogen-mediated gut-vaginal axis and substantial taxonomic overlap between these niches (56, 65). Integrating gut and cervicovaginal microbiome assessment in future HSIL treatment-response cohorts could clarify whether interventions targeting either or both compartments confer benefit.

### 4.3 Limitations of the evidence base

Several methodological limitations temper confidence in these conclusions and constrain immediate clinical translation. Sample sizes in the prospective LEEP/conization-microbiome cohorts identified were uniformly small (typically 26–108 participants), limiting statistical power to detect modest effect sizes and precluding robust subgroup analysis by HPV genotype, lesion grade, or menopausal status (22–24, 26). Microbial profiling methodology varied substantially across studies — including Nugent-score microscopy, targeted qPCR microarray, 16S rRNA amplicon sequencing of differing hypervariable regions, and shotgun metagenomics — limiting direct comparability and pooling of findings, a limitation explicitly acknowledged in the existing systematic review and meta-analysis of Lactobacillus therapy (62). Follow-up intervals after treatment were generally short (3–12 months), insufficient to capture later recurrence or long-term oncological outcomes, and few studies adjusted for potential confounders such as sexual activity, antibiotic exposure, contraceptive method, smoking, or HIV status, all of which independently influence both the CVM and HPV natural history (24, 46).

Population heterogeneity is a further constraint: CST distribution and its clinical correlates differ across ethnic and geographic populations, as demonstrated in Latina, Chinese, Korean, Kazakh, Malawian, and Canadian cohorts included in this review (15, 16, 23, 26, 32, 46), such that findings from any single population may not generalize globally, and pooled or multi-ethnic prospective cohorts are needed. Finally, most mechanistic evidence linking specific bacterial metabolites or species to E6/E7 modulation derives from *in vitro* cell-line experiments (notably SiHa cells) (34, 47), which, while hypothesis-generating, require confirmation *in vivo* and in clinical cohorts before mechanistic claims can be considered established.

### 4.4 Strengths and limitations of this review

This scoping review’s principal strength is its comprehensive mapping of a genuinely cross-disciplinary literature — spanning microbiology, immunology, gynecologic oncology, and biomedical informatics — using a transparent, PRISMA-ScR-concordant methodology (30), allowing identification of translational gaps (absence of interventional trials timed around excisional treatment; absence of treatment-response-specific machine-learning models) that would not be visible from narrower systematic reviews restricted to a single study design or outcome. As a scoping rather than systematic review, however, this synthesis did not perform formal risk-of-bias appraisal or quantitative pooling of effect estimates, consistent with the scoping review methodology deployed (27, 28, 31), and findings should therefore be interpreted as descriptive of the evidence landscape rather than as a definitive, bias-adjusted effect estimate for any single association. The search, while structured and iterative across multiple platforms, did not follow a fully systematic multi-database protocol with duplicate independent screening, and language restriction to English-language sources may have excluded relevant non-English literature, particularly from China, where a substantial proportion of the primary LEEP/conization-microbiome literature originates.

### 4.5 Future research priorities

- Adequately powered, multi-ethnic prospective cohorts assessing CVM composition immediately before, and at multiple time points after, LEEP or conization, with hrHPV clearance/persistence and histological recurrence as co-primary endpoints and standardized microbial profiling methodology.
- Randomized controlled trials of intravaginal Lactobacillus (particularly L. crispatus strain) transplantation as an adjunct to excisional HSIL treatment, powered for post-treatment hrHPV clearance at 6–12 months.
- Development and external validation of machine-learning or multi-omics predictive models specifically trained on post-treatment outcome data, rather than lesion presence/severity alone, incorporating microbiome, metabolome, and immunoproteome features.
- Integrated gut-cervicovaginal microbiome studies in the HSIL treatment setting, informed by chemoradiotherapy-era findings on the gut-vaginal axis and gut diversity’s association with treatment response in invasive disease.
- Mechanistic studies extending *in vitro* E6/E7-modulation findings into *in vivo* and clinical validation, and characterization of age- and menopause-related CVM shifts as effect modifiers of treatment response.
- Standardization of CST classification, sequencing methodology, and outcome definitions across studies to enable future systematic review and meta-analysis, and building on the existing registered protocol for post-treatment CVM changes.

## 5 Conclusion

The cervicovaginal microbiome (CVM) is an increasingly well-supported and biologically plausible predictor of hrHPV clearance and response to excisional and ablative treatment of HSIL. *Lactobacillus crispatus* dominance is consistently associated with favorable outcomes across observational and mechanistic studies and, to a more limited extent, interventional evidence. In contrast, CST-IV dysbiosis and bacterial vaginosis are associated with hrHPV persistence and treatment failure, potentially through lactic acid- and metabolite-mediated regulation of HPV oncogene expression and local immune responses. Excisional treatment itself substantially reshapes the CVM, suggesting that the post-treatment trajectory toward Lactobacillus dominance may provide greater prognostic value than pretreatment microbiome status alone. Nevertheless, small sample sizes, methodological heterogeneity, limited follow-up, and, critically, the absence of trials and predictive models specifically focused on the peri-treatment period currently preclude routine integration of CVM profiling or microbiome-directed interventions into HSIL management. Prospective, adequately powered, methodologically standardized studies centered on the excisional treatment window are therefore the most important and clinically relevant next step for establishing the predictive and therapeutic value of the CVM.
